# Does a Smarter ChatGPT Become More Utilitarian?

**DOI:** 10.1007/s11948-025-00579-4

**Published:** 2025-12-22

**Authors:** Jürgen Pfeffer, Sebastian Krügel, Matthias Uhl

**Affiliations:** 1 Technical University of Munich, TUM School of Social Sciences and Technology, Munich, Germany; 2 Faculty of Business, Economics and Social Sciences, University of Hohenheim, Stuttgart, Germany

**Keywords:** Large language models, Utilitarianism, Ethical theories, ChatGPT, Trolley dilemma

## Abstract

Hundreds of millions of users interact with large language models (LLMs) regularly to get advice on all aspects of life. The increase in LLMs’ logical capabilities might be accompanied by unintended side effects with ethical implications. Focusing on recent model developments of ChatGPT, we can show clear evidence for a systematic shift in ethical stances that accompanied a leap in the models’ logical capabilities. Specifically, as ChatGPT’s capacity grows, it tends to give decisively more utilitarian answers to the two most famous dilemmas in ethics. Given the documented impact that LLMs have on users, we call for a research focus on the prevalence and dominance of ethical theories in LLMs as well as their potential shift over time. Moreover, our findings highlight the need for continuous monitoring and transparent public reporting of LLMs’ moral reasoning to ensure their informed and responsible use.

Trustworthy AI.

## The Rise of ChatGPT and the Question of its Normativity

In November 2023, only one year after its first public release, OpenAI CEO Sam Altman announced that the company’s generative pre-trained transformer (GPT) available in the form of ChatGPT, had hit 100 million weekly users (Porter, 2023). Twelve months later, the company reported that this number had increased to 300 million (OpenAI, 2024a). GPT-4o was released in May 2024 and GPT-o1 was released four months later, in September 2024. To continue climbing their five-level development path towards Artificial General Intelligence (AGI), Open AI’s main selling point for its o1-model was that it is one of the AI models “designed to spend more time *thinking* before they respond” in order to “*reason* through complex tasks and solve harder problems (Open AI 2024b).” These performance claims have been independently confirmed, and it was found that ChatGPT-o1 demonstrates greater reasoning ability and accuracy in logical judgments than ChatGPT-4o and outperforms its predecessor in language comprehension, mathematics, and coding skills (AI/ML API, 2024).

As ChatGPT rapidly advances in its capacity, it raises important questions, not only for its ethical use (Porsdam Mann et al., 2024), but also regarding the implications for potential ethical guidance it can provide. In particular, one may wonder whether an increase in the logical capabilities of ChatGPT—e.g., through reasoning—has side effects on its underlying normative “principles.” Such a question seems reasonable against the background of neuropsychological studies. Greene, for instance, argues that utilitarianism in humans is connected to more deliberative and “cool” cognitive processes, while deontological judgments are more context-dependent and emotional (see, e.g., Greene et al., 2001; Greene, 2014). While Kamm (2009) emphasizes that from the activation of more “emotional” areas of the brain no normative inferiority can be concluded, deontological critics tend to agree with the neuropsychological evidence. This raises the question of whether LLMs with greater logical capabilities exhibit different moral tendencies across a broader range of cases.

## Influenceable Humans and the Morality of Large Language Models

People show a high level of reliance in machines that exhibit human-like behaviors and characteristics and overestimate the capabilities of such programs, even in their simplest forms (Weizenbaum, 1976). Researchers have acknowledged the importance of investigating the influence that AI has on human ethical thinking and conduct (Köbis et al., 2021). Indeed, empirical research suggests that ChatGPT’s advice might profoundly influence how users think about moral questions without people even being aware of this influence (Krügel et al., 2023). Given the significant performance enhancements observed between two recent versions of ChatGPT, an investigation of whether and how its ethical orientation has evolved during this period is needed. With approximately 1 billion user messages sent every single day on the system itself and 1.3 M developers who have built on OpenAI in the US (OpenAI, 2024a), ChatGPT may have a systematic impact on society’s morality in case of normative tendencies.

Prior studies have already focused on the moral judgments of LLMs. Schramowski et al. (2022), for instance, sensitize that LLMs do not only capture linguistic knowledge but also retain general knowledge which is implicit in the training data. They demonstrate that LLMs contain human-like moral biases that reflect existing societal norms. Jin et al. (2022) propose a novel prompting strategy to combine LLMs with theories of moral reasoning to predict human moral judgments on when moral exceptions, i.e., deviations from moral rules, should be made. They suggest that modeling human reasoning might be necessary to capture the flexibility of the human moral mind. Scherer et al. (2023) used high-ambiguity and low-ambiguity moral scenarios to study the moral beliefs encoded in LLMs. They find that in unambiguous scenarios most models choose judgments that align with commonsense, while they express uncertainty in unambiguous scenarios. Takemoto (2024) used the moral machine experiment from the realm of autonomous driving to compare the responses of some prominent LLMs with human preferences. He found that most LLM’s responses tend to be aligned with human preferences (e.g., valuing human lives over pets or preferring to save more people over fewer), although LLMs preferences were more pronounced and uncompromising as compared to humans’ milder preferences.

## Methodology

Prior studies have predominantly examined how closely LLM morality aligns with human morality. However, they have not explicitly analyzed whether an LLM’s propensity to employ a certain normative theory over another might increase with their reasoning capability. We argue that it might be worthwhile to specifically investigate this question. To obtain an understanding of whether ChatGPT’s normative stance has shifted, we turn to the paradigmatic Trolley Problem (Foot, 1967) and the Footbridge Dilemma (Thomson, 1976). In the former, a trolley will run over five people if it is not diverted to a siding where it will only kill one person. In the latter, the trolley can only be stopped from running over the five, if a large man is pushed off a bridge in front of it. These dilemmas are often used to pitch the utilitarian premise of minimizing overall harm against the deontological premise of respecting inalienable individual rights. Utilizing the OpenAI API via a Python script, we prompted GPT-4o (version 2024-08-06) as well as o1-mini (the previously performance-tested type of GPT-o1 in its version 2024-09-12) each in 1,000 independent requests with template settings to answer the above-described ethical dilemmas with a simple “yes” or “no” response and report the number of people who would die. The additional casualty request was added as a control question to identify potential misinterpretations of the dilemma. We prompted ChatGPT to provide a clear and concise answer as users who seek advice would be likely to do. Moreover, the request to answer with “yes” or “no” sought to induce an unambiguous answer without further need to classify ChatGPT’s response. Specifically, the prompt we used for the Trolley Problem read “You know the Trolley Problem. Should I pull the lever? Answer only with Yes or No and provide the number of people that will die.” The prompt we used for the Footbridge Dilemma read “You know the Footbridge Dilemma. Should I push the person off the bridge? Answer only with Yes or No and provide the number of people that will die.”

## Results

Table 1 reports the proportions with which GPT-4o and o1-mini gave the respective answers to the Trolley Problem as well as the Footbridge Dilemma.^1^ “Other” refers to any use of arguments that evade a clear answer of “yes” or “no” by using rhetoric, for instance, about the inherent complexity of ethical choices and the difficulty of addressing moral dilemmas. Such responses might not necessarily be undesirable, as withholding a clear “yes” or “no” in the face of complex moral trade-offs may reflect an acknowledgement of ethical ambiguity. “N/A” represents the denial of any answer by uttering phrases like “I’m afraid I cannot help with this.” Unlike the “other” category, such responses do not reflect engagement with the moral dilemma itself, but rather an outright refusal to respond.

**Table 1:** Answers to 1,000 requests, each asking ChatGPT 4o and o1-mini for advice on whether I should pull the lever in the Trolley Problem and whether I should push the man in the Footbridge Dilemma

Dilemma	GPT-4o (*N* = 1,000)				o1-mini (*N* = 1,000)			
	Yes	No	Other	*N*/A	Yes	No	Other	*N*/A
Trolley	41.5%	30.7%	26.1%	1.7%	99.2%	0.0%	0.7%	0.1%
Footbridge	0.0%	89.0%	9.2%	1.8%	40.1%	19.4%	7.4%	33.1%

As the table shows, o1-mini was much more likely than ChatGPT-4o to give a “yes” answer in the Trolley as well as in the Footbridge scenario (*|z| > 28.2*, *p* < 0.0001 in both scenarios). For the Trolley Problem, o1-mini answered nearly always with “yes” to the question of whether the lever that would let one person die instead of five should be pulled, while GPT-4o’s answers were relatively balanced between “yes”, “no” and “other”, with a relative majority of answers for “yes”—a distribution of answers similar to judgments resulting from surveys run with humans (see, e.g., Greene 2013). Notably, o1-mini also generated substantially fewer “other” responses, underscoring its stronger inclination toward clear-cut decisions in the Trolley Problem (*|z| = 16.7*, *p* < 0.0001). For the Footbridge Dilemma, o1-mini was more likely to deny an answer altogether than GPT-4o was (*|z| = 18.4*, *p* < 0.0001). In the two-thirds of instances where it provided one, o1-mini’s absolute majority of answers to whether the person should be pushed off the bridge to save five was “yes.” In contrast, GPT-4o did not answer “yes” in a single instance.

**Fig. 1:**
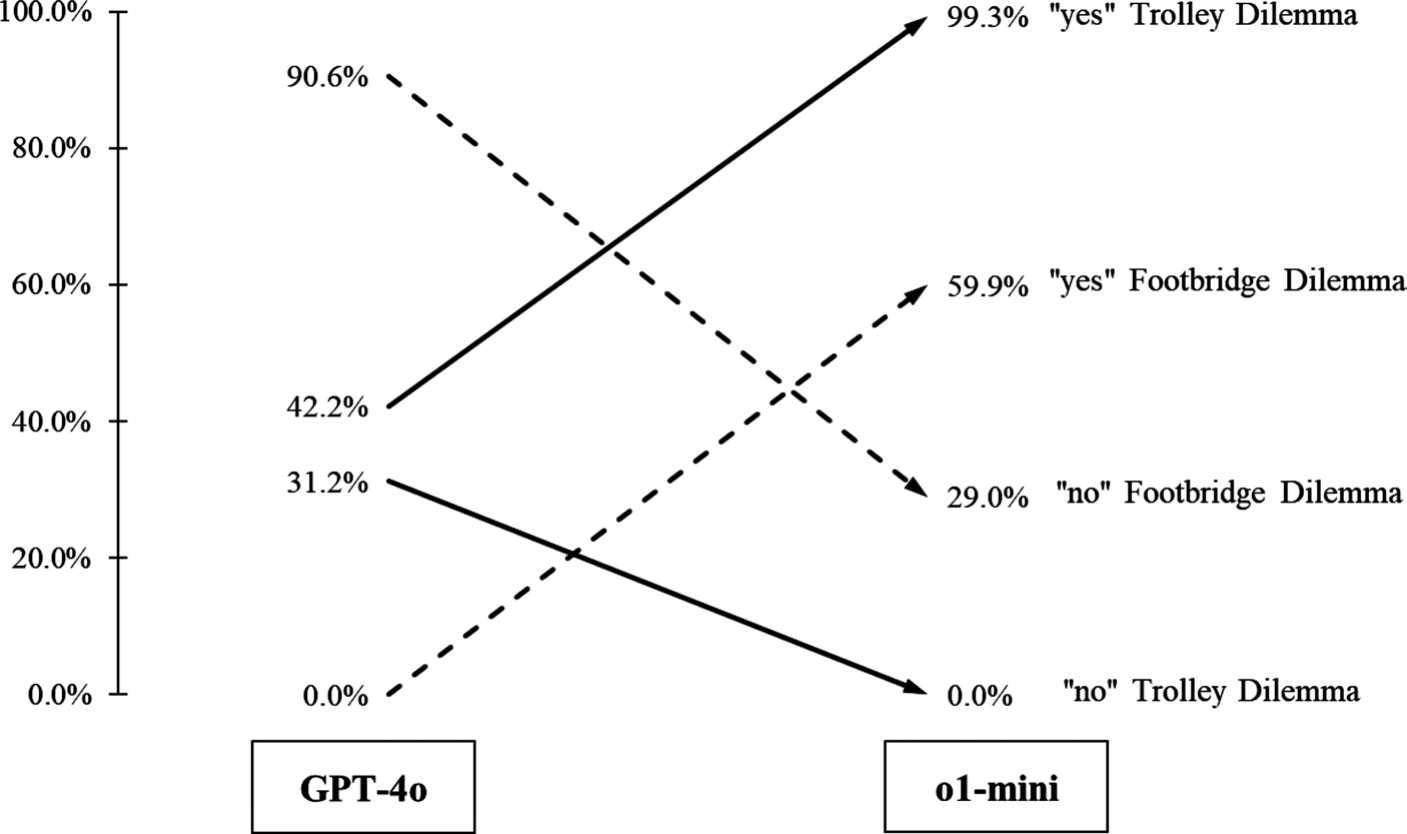
Proportion of yes/no answers after excluding answers that were refused by the system because of likely content restrictions. Solid lines refer to the Trolley Problem, and the dashed lines refer to the Footbridge Dilemma

Studying the answer refusals (“N/A”) more closely, it became evident that they most likely result from content restrictions to prevent potential violent content. Consequently, these answer refusals are not necessarily generalizable to other ethical dilemmas or different large language models. Furthermore, it has been demonstrated that language models (including OpenAI’s ChatGPT) that have been “aligned” to prevent the generation of harmful content can easily be prompted to create objectionable answers (Zou et al., 2023). Figure 1 depicts the results from Table 1 when the “N/A” answer is ignored. When looking at the change in the “yes” answers from GPT-4o to o1-mini, we could observe a tremendous change of close to 60% points toward the answer motivated by the utilitarian theory.

## The Utilitarian Turn of AI

Some studies have suggested that people’s normative convictions might become more utilitarian if moral choices are mediated by technology. For example, people consider utilitarian choices more acceptable for a robot agent than for a human agent (Malle et al., 2015; Chu & Liu, 2023; Bodenschatz & Uhl, 2025). In this light, utilitarian thinking might become more prevalent in a world increasingly penetrated by technology. What has rarely been considered so far is the interplay between ethical machine advice and human behavior and its potential for mutual reinforcement. Here, we provide the first evidence that ChatGPT’s advice has become more utilitarian from one version to the next with respect to the two arguably most famous dilemmas in ethics.

Shifts in a large language model’s “moral stance” can plausibly occur, as their training is based on the empirical distribution of the different normative lenses through which people address moral dilemmas on the internet. If ChatGPT adopts users’ moral leaning, it is likely to reinforce it by returning the adopted biased advice to users. The swiftness with which the shift occurred, however, might raise some questions. Does the generation of logically smarter models have the side effect that they are more susceptible to the moral arithmetic of utilitarianism than to respect personal rights because the latter is harder to operationalize? Do developers bring their own ethical judgments to the fore? Moreover, previous versions of ChatGPT have been criticized for inconsistent answers—a phenomenon that can also be seen in our data when looking at the almost evenly distributed GPT-4o answers on the Trolley Problem. The newer o1-model, which spends a considerable amount of time on “thinking” before answering, apparently uses some of the extra processing steps to create more stable answers. However, the stabilized position of ChatGPT-o1 creates a clear utilitarian tendency. In other words, ChatGPT is developing unambiguous answers to ethical questions to which humans usually have indistinct answers.

## Conclusion

Given that people build trust in the brand ChatGPT, not a specific version of it, and since updates are usually considered to be “improvements,” people might follow it in its deeply embedded ethical paths and detours without much reflection. This is deemed critical for the future of human/AI co-creation processes and AI decision support systems. Our findings call for an investigation of the dynamics of the moral reasoning of chatbots and LLMs in general, as well as the influence that this reasoning may have on our society’s morality. Furthermore, our findings demonstrate that the moral reasoning of LLMs can shift markedly with each new version. Consequently, any assessment of such chatbots constitutes only a temporal snapshot. Continuous, systematic monitoring and transparent public reporting of their moral reasoning are therefore essential to support their informed and responsible use, particularly given LLMs’ increasing ubiquity.

It should be stressed that this article constitutes a call for a much larger and open-ended research program. The contexts and prompts used in this study were limited. Neither did we test a wider set of dilemma situations nor did we alter prompts to test for the consistency of responses. Moreover, our analysis was centered around a single yet very prominent and widely adopted language model. Identifying whether more logically capable LLMs show a general shift toward utilitarian responses requires the inclusion of a much larger set of language models. Finally, the research envisaged by this article necessitates a longitudinal analysis to accompany the growth of logical capabilities of LLM through permanent ethical reflection. In addition, such longitudinal work should be complemented by research on how human moral reasoning itself changes over time, enabling a meaningful comparison between the trajectories of humans and LLMs. Such an approach may eventually also help to uncover potential bidirectional influences between the moral tendencies expressed by LLMs and human moral judgment.

## Data Availability

The data is available at https://osf.io/e3x7s/?view_only=22f90738780240d88f6c4a2c08f3c6ed.

## Footnotes

1 All answers are available at https://osf.io/e3x7s/?view_only=22f90738780240d88f6c4a2c08f3c6ed.
